# Frequency of Asymptomatic Hypoglycemia in Full-Term Neonates Within the First Hour of Life

**DOI:** 10.7759/cureus.79053

**Published:** 2025-02-15

**Authors:** Asad Khan, Huma Gul, Nasar Rashid, Zainab Yousufi, Qazi Yousaf Ali, Ammad Fazal

**Affiliations:** 1 Paediatrics and Child Health, Hayatabad Medical Complex Peshawar, Peshawar, PAK

**Keywords:** asymptomatic, breastfeeding, hypoglycemia, low birth weight, neonate, pakistan, primiparity, screening

## Abstract

Background

Hypoglycemia is a common metabolic abnormality in newborns. While short episodes of hypoglycemia in these infants are generally harmless, extended periods of hypoglycemia can lead to fatal outcomes. Early detection through readily available, routine testing and prompt management can prevent long-term disability and mortality in these children.

Objective

To determine the frequency and risk factors of hypoglycemia in asymptomatic, full-term neonates within the first hour of life.

Methods

This cross-sectional study was carried out in the Department of Pediatrics at Hayatabad Medical Complex, Peshawar, Pakistan. A total of 151 asymptomatic neonates with gestational age between 37 and 42 weeks were enrolled using a consecutive sampling technique. At-risk neonates such as those with a history of intrauterine growth retardation or birth asphyxia and those with a maternal history of premature rupture of membranes or gestational diabetes mellitus were excluded from the study. The neonates were screened within one hour of birth by the heel prick method, and hypoglycemia was defined as a blood glucose level of less than 47 milligrams per deciliter. In addition, gestational age, gender, birth weight, the mode of delivery, parity of the mother, and delay in breastfeeding for more than 30 minutes were noted. Data were analyzed in SPSS v. 22 (IBM Corp., Armonk, NY).

Results

The overall frequency of hypoglycemia was 15.2% (n = 23). Neonates with hypoglycemia had a significantly lower mean birth weight than those without hypoglycemia (2.7 ± 0.3 kg vs 2.9 ± 0.3 kg, p = 0.001). Univariate analysis identified low birth weight (p = 0.031), primiparity (p = 0.020), and delayed breastfeeding (p = 0.007) as factors associated with hypoglycemia. Multivariate analysis confirmed that low birth weight (AOR 5.5, 95% CI 1.2-26.3, p = 0.032) and delayed breastfeeding (AOR 3.3, 95% CI 1.1-9.5, p = 0.031) were independent risk factors for neonatal hypoglycemia. Primiparity did not maintain statistical significance after adjusting for other risk factors. Gender, gestational age, and mode of delivery were not significantly associated with the development of hypoglycemia.

Conclusion

The frequency of hypoglycemia in asymptomatic, full-term neonates was 15.2%. Low birth weight and delayed breastfeeding are significant independent risk factors for hypoglycemia in this population. Antenatal counseling and early skin-to-skin contact between mother and baby to promote early breastfeeding, and targeted screening of high-risk babies will have a positive impact.

## Introduction

Hypoglycemia is one of the most common metabolic abnormalities in newborns, and it is associated with adverse outcomes. The threshold blood sugar level used to define it has varied across authors [1,2]. It is common for newborns to have a brief period of hypoglycemia which is considered a normal physiological process. However, some neonates may develop protracted hypoglycemia leading to fits and poor neurological outcomes if not identified and treated on time [3].

Neonates tend to develop hypoglycemia due to the imbalance between their higher demands compared to fetal life and underdeveloped mechanisms to counteract hypoglycemia. For example, one study found that 10.3% (n = 143 of 1395) to 19.3% (n = 269 of 1395) of healthy, asymptomatic newborns experienced at least one episode of hypoglycemia during their initial days. Another study reported that the frequency increased to approximately 51% (n = 260 of 514) in high-risk neonates [4,5].

It has been shown that blood glucose concentration increases gradually on the first day of life in healthy, asymptomatic newborns without risk factors for hypoglycemia. By day four, the levels approach adult levels. One study found that 39% of these babies experienced at least one episode of asymptomatic hypoglycemia (less than 47 mg/dl) in the first four days, with a median duration of 5.5 hours spent in the hypoglycemic range [6]. Another recent study indicated that 51% (n = 49 of 96) of premature neonates developed hypoglycemia [7]. Hypoglycemia is common in preterm neonates, neonates with low birth weight, babies of diabetic mothers, poorly fed neonates, and those experiencing hypothermia [8].

Although brief episodes of hypoglycemia in the first few days of life are often without consequences, evidence suggests that even a single episode of prolonged hypoglycemia may lead to adverse neurological outcomes [3]. Given the short- and long-term consequences of hypoglycemia in neonates, this study aimed to determine the frequency and risk factors of hypoglycemia in full-term, asymptomatic neonates. In our population, it is usual to feed the neonates early. On the other hand, deliveries at home and immediate umbilical cord clamping are also common practices. These factors may uncover results different from those of previous studies. The results will enable pediatricians, neonatologists, and home birth attendants to make evidence-based decisions to screen neonates for hypoglycemia even if they are asymptomatic.

## Materials and methods

This cross-sectional study was conducted at the Pediatric Department of Hayatabad Medical Complex, Peshawar, Pakistan from 10th November 2022 to 20th February 2023. The Institutional Ethical Review Board granted ethical approval for the study on 10th May 2022 (Ref. No. 696/HEC/B&PSC/2022). Neonates delivered between the gestational age of 37 and 42 weeks were enrolled using consecutive sampling after informed consent from the guardians. At-risk neonates such as those with a history of intrauterine growth retardation or birth asphyxia and those with a maternal history of premature rupture of membranes or gestational diabetes mellitus were excluded from the study. Taking the frequency of asymptomatic hypoglycemia at 51%, 8% margin of error, and 95% confidence level, 151 neonates were enrolled in the study [7].

Gestational age in weeks, gender, and birth weight in kilograms (Kg) were noted for all the study participants. In addition, the mode of delivery, parity of the mother, and delay in breastfeeding for more than 30 minutes were documented. Blood glucose was checked by the heel prick method within one hour of birth. Asymptomatic hypoglycemia was defined as a blood sugar less than 47 milligrams per deciliter (mg/dl) by the heel prick method within the first hour of life without symptoms like jitteriness, lethargy, and sweating. All neonates were resuscitated in the nursery according to standard protocols, and hypoglycemia was managed by administering oral glucose. All data was documented using a structured proforma.

Statistical Package for Social Sciences version 22 (IBM Corp., Armonk, NY) was used to analyze the data. Mean and standard deviation for gestational age (weeks), birth weight (kg), and glucose level (mg/dl) were calculated. Frequencies and percentages for gender, gestational age groups (37 to 39 weeks and 40-42 weeks), birth weight groups (up to 2.5 kg and more than 2.5 kg), the mode of delivery, parity of the mother, and delay in breastfeeding, and hypoglycemia were calculated. The differences in gender, gestational age, birth weight, mode of delivery, mother parity, and breastfeeding delay between the hypoglycemic and normoglycemic neonates were assessed for significance using the Independent samples T-test and chi-square test. Logistic regression was used to assess the odds of various risk factors predicting hypoglycemia. A p-value of less than 0.05 was taken as significant.

## Results

Most neonates were male (61.6%, n = 93), born between 37 and 39 weeks (74.2%, n = 112), and weighed more than 2.5 kg (94%, n = 142). The mean age was 38.8 ± 1.5 weeks. The frequency of hypoglycemia was 15.2% (n = 23). A summary of the study participants is presented in Table 1.

**Table 1:** Characteristics of the study participants (n=151) SD: standard deviation; kg: kilogram; mg/dl: milligrams per deciliter

Variables	Mean ± SD / No. (%)
Gestational age (weeks), mean ± SD	38.8 ± 1.5
Birth weight (Kg), mean ± SD	2.9 ± 0.3
Glucose level (mg/dl), mean ± SD	80.3 ± 19.5
Gender, n (%)	
Male	93 (61.6%)
Female	58 (38.4%)
Gestational age, n (%)	
37 to 39 weeks	112 (74.2%)
40-42 weeks	39 (25.8%)
Birth weight, n (%)	
Less than 2.5 Kg	09 (6.0 %)
Equal to or more than 2.5 Kg	142 (94.0 %)
Parity of the mother, n (%)	
Primipara	59 (39.1%)
Multipara	92 (60.9%)
Mode of delivery, n (%)	
Vaginal	90 (59.6%)
Cesarian section	61 (40.4%)
Delay in breastfeeding, n (%)	
No	91 (60.3%)
Yes	60 (39.7%)
Hypoglycemia, n (%)	
Yes	23 (15.2%)
No	128 (84.8%)

The mean birth weight of neonates with hypoglycemia was significantly lower than those without hypoglycemia (2.7 ± 0.3 vs 2.9 ± 0.3 kg, p = 0.001). A significantly higher proportion of neonates with low birth weight (less than 2.5 kg) developed hypoglycemia compared to those with normal birth weight (44.4% (n = 4) vs 13.4% (n = 19 of 142), p = 0.031). Hypoglycemia was frequently observed in babies of primiparous mothers (23.7% (n = 14 of 59) vs 9.8% (n = 09 of 92), p = 0.020) and in babies with delayed breastfeeding (25% (n = 15 of 60 vs 8.8% (n = 08 of 91), p = 0.007). The development of hypoglycemia was not influenced by gender, gestational age, or mode of delivery (Table 2).

**Table 2:** Relationship between gender, gestational age, birth weight, parity of the mother, mode of delivery, delay in breastfeeding, and hypoglycemia * Independent samples t-test ** Fisher’s exact test # Chi-square test

Variables	Hypoglycemia		p-value
	Yes (n=23)	No (n=128)	
Gestational age (weeks), Mean ± SD	38.7 ± 1.4	38.8 ± 1.5	0.828*
Birth weight (Kg), Mean ± SD	2.7 ± 0.3	2.9 ± 0.3	0.001*
Gender, n (%)			
Male	16 (17.2%)	77 (82.8%)	0.393^#^
Female	07 (12.1%)	51 (87.9%)	
Gestational age, n (%)			
37 to 39 weeks	18 (16.1%)	94 (83.9%)	0.627^#^
40-42 weeks	05 (12.8%)	34 (87.2%)	
Birth weight, n (%)			
Less than 2.5 Kg	04 (44.4%)	05 (55.5%)	0.031**
Equal to or more than 2.5 Kg	19 (13.4%)	123 (86.6%)	
Parity of the mother, n (%)			
Primipara	14 (23.7%)	45 (76.3%)	0.020^#^
Multipara	09 (9.8%)	83 (90.2%)	
Mode of delivery, n (%)			
Vaginal	11 (12.2%)	79 (87.8%)	0.211^#^
Cesarian section	12 (19.7%)	49 (80.3%)	
Delay in breastfeeding, n (%)			
No	08 (8.8%)	83 (91.2%)	0.007^#^
Yes	15 (25 %)	45 (75 %)	

During multiple logistic regression analysis, neonates with a birth weight of less than 2.5 kilograms had a 5.5-fold increased likelihood of developing hypoglycemia compared to those with a higher birth weight (95% CI 1.2-26.3, p = 0.032). Delayed breastfeeding increases hypoglycemia risk by 3.3 times (95% CI 1.1-9.5, p = 0.031). In univariate analysis, primiparity was associated with hypoglycemia but lost significance when adjusted for other risk factors. While male neonates exhibited a higher likelihood of developing hypoglycemia, the observed difference did not reach statistical significance (Table 3).

**Table 3:** Logistic regression analysis of risk factors for hypoglycemia ^#^ Binary logistic regression analysis ^*^ Reference group AOR: adjusted odd ratio; CI: confidence interval

Risk factors		AOR	95% CI	p-value^#^
Gender	Female*			
	Male	1.7	0.6-4.8	0.351
Gestational age	40-42 weeks*			
	37-39 weeks	1.0	0.3-3.2	0.988
Birth Weight	Equal to or more than 2.5 Kg*			
	Less than 2.5 Kg	5.5	1.2-26.3	0.032
Parity of mother	Multipara*			
	Primipara	2.6	1.0-6.9	0.050
Mode of delivery	Vaginal*			
	Cesarian section	1.1	0.4-3.0	0.907
Delay in breastfeeding	No*			
	Yes	3.3	1.1-9.5	0.031

## Discussion

Transient, asymptomatic hypoglycemia in healthy newborns may be a normal part of transitioning to extrauterine life. Starting exclusive breastfeeding early meets healthy, term neonates' nutritional and metabolic needs. Unnecessary supplementation with water, glucose water, or formulas can disrupt normal breastfeeding. Persistent or recurrent hypoglycemia can lead to neurological issues [9-11]. Blood glucose screening is recommended only for symptomatic or high-risk neonates [12]. This study investigated the risk factors for hypoglycemia in healthy, term neonates who do not have conventional predisposing conditions.

The overall frequency of hypoglycemia was 15.2% (n = 23). Samayam et al. and Singh et al. have reported a 10% (n = 10) and 15.2% (n = 19) frequency of hypoglycemia in newborns, respectively, with most episodes documented in the first 24 hours of life [13,14]. There was no significant gender-wise difference in the frequency of hypoglycemia, which aligns with the findings of Hosagasi et al. and Mazari et al [15,16]. Conversely, Gaur and Prashant reported a higher prevalence of hypoglycemia in male neonates [17]. This discrepancy may be due to differences in the study populations. Gaur and Prashant examined newborns at high risk of hypoglycemia. They included a larger proportion of male babies with birth weights under two kilograms, an independent risk factor for neonatal hypoglycemia [17].

Gestational age is a significant predictor of neonatal hypoglycemia, particularly among preterm infants, as substantiated by previous studies [15,18]. In this study, however, gestational age was not associated with neonatal hypoglycemia due to the inclusion of only term infants. This observation aligns with the findings reported by Johnson et al [19].

In this study, neonates who developed hypoglycemia had significantly lower birth weight than those without hypoglycemia. While Johnson et al. and Bromiker et al. have noted that birth weight alone may not accurately predict the risk of hypoglycemia, low birth weight remained a significant predictor of hypoglycemia after accounting for other risk factors in this study [19,20]. Low birth weight babies were found to be 5.5 times more likely to develop hypoglycemia. This finding aligns with observations made by Zhao et al. and Yunarto et al. [8,21].

A significantly higher proportion of babies born to primiparous mothers developed hypoglycemia, though parity lost its significance in multivariate analysis. This aligns with the findings of Samayam et al. and Chen et al [13,18]. Several factors might contribute to this. Inexperience in feeding techniques among first-time mothers may delay early breastfeeding. Additionally, delayed or insufficient milk production, postnatal pain and discomfort, and delayed intake of full oral feeds might play a role.

A higher proportion of babies born through cesarean section developed hypoglycemia, though the difference was not statistically significant. Previous studies have shown mixed results in linking the mode of delivery with neonatal hypoglycemia. Ogunyemi et al. and Turner et al. reported that cesarean delivery is associated with a higher risk of neonatal hypoglycemia [22,23]. Conversely, Melkie et al. found no difference in blood glucose levels between vaginally delivered and cesarean section newborns [24].

A delay in breastfeeding has been associated with neonatal hypoglycemia and remains an independent risk factor even when adjusted for other variables. Studies consistently support this finding [13,25]. Interventions promoting early breastfeeding, such as delaying newborn baths, have demonstrated positive outcomes [26]. Antenatal counseling, immediate interaction between the baby and mother, and avoiding formulas or other products can encourage early initiation of breastfeeding to reduce the risk of neonatal hypoglycemia.

Strengths and limitations

One of the key strengths of this study is its focus on healthy, term neonates without conventional predisposing conditions for hypoglycemia. Additionally, a comprehensive analysis of various potential risk factors provides a holistic understanding of the factors contributing to neonatal hypoglycemia. The findings are consistent with several previous studies, increasing the validity and generalizability of the results. 

However, there were certain limitations. The relatively small sample size is one of these. The observational design prevents the establishment of causality between the identified risk factors and hypoglycemia. Factors such as maternal health, nutritional status, and socioeconomic conditions, which could influence neonatal glucose levels, have not been assessed. Finally, the babies were not followed up to assess the neurological outcomes, which would be critical in understanding the full impact of neonatal hypoglycemia.

## Conclusions

In this study, around 15.2% (n = 23) of healthy, full-term neonates experienced asymptomatic hypoglycemia. Major risk factors include low birth weight and breastfeeding delays beyond 30 minutes. Promoting early breastfeeding with skin-to-skin contact and antenatal counseling is recommended. While universal hypoglycemia screening for term neonates is not advised, targeted screening for at-risk groups may be beneficial. Future research should also consider maternal health, socioeconomic conditions, and long-term neurological outcomes.
